# MRSA Profiles Reveal Age- and Gender-Specificity in a Tertiary Care Hospital: High Burden in ICU Elderly and Emerging Community Patterns in Youth

**DOI:** 10.3390/microorganisms13051078

**Published:** 2025-05-06

**Authors:** Kamaleldin B. Said, Khalid Alshammari, Ruba M. Elsaid Ahmed, Fawwaz Alshammari, Ahmed H. Jadani, Ihab Rakha, Salem A. Almijrad, Anwar E. Almallahi, Bader Alkharisi, Naif M. Altamimi, Tarig Mahmoud, Nada A. Abozaid, Amal D. Alshammari

**Affiliations:** 1 Department of Pathology, College of Medicine, University of Ha’il, Ha’il 55462, Saudi Arabia; rm.ahmed@uoh.edu.sa (R.M.E.A.); s202005564@uoh.edu.sa (S.A.A.);; 2 Department of Internal Medicine, College of Medicine, University of Ha’il, Ha’il 55462, Saudi Arabia; 3 Department of Dermatology, College of Medicine, University of Ha’il, Ha’il 55462, Saudi Arabia; 4 Departments of Clinical Microbiology, King Khalid Hospital, Ha’il 55462, Saudi Arabia; 5 Department of Obstetrics and Gynecology, College of Medicine, University of Ha’il, Ha’il 55476, Saudi Arabia; tm.ahmed@uoh.edu.sa; 6 Department of Health Administration, College of Public Health and Health Informatics, University of Ha’il, Ha’il 55462, Saudi Arabia; 7 Department of Family and Community Medicine, College of Medicine, University of Ha’il, Ha’il 55462, Saudi Arabia

**Keywords:** *Staphylococcus aureus*, MRSA, MSSA, tertiary care hospital, antibiotic resistance, ICU

## Abstract

Methicillin-resistant *Staphylococcus aureus* (MRSA) is a devastating global health concern. Hypervirulent strains are on the rise, causing morbidities and mortalities worldwide. In tertiary care hospitals, critically ill patients, those undergoing invasive procedures, and pediatric and geriatric patients are at risk. It is not fully clear how strains adapt and specialize in humans and emerge despite the well-established commonality of the *S. aureus* genome from humans and animals. This study investigates the influence of age-, gender-, and source-specific profiles (clinical, intensive care unit (ICU vs. non-ICU)) on the evolution of hospital-associated (HA)-MRSA versus community-associated (CA)-MRSA lineages. A total of 253 non-duplicate *S. aureus* isolates were obtained from May 2023 to March 2025. The patients were stratified by age and gender in ICUs and non-ICUs. Standard microbiology methods and Clinical and Laboratory Standards Institute (CLSI) guidelines were used for identification and susceptibility testing, with cefoxitin and oxacillin disk diffusions and molecular diagnosis confirming MRSA. Mann–Whitney U and Chi-square tests assessed the demographic distributions, clinical specimen sources, and MRSA/methicillin-sensitive *S. aureus* (MSSA) prevalence. Of 253, 41.9% originated from ICUs (71% male; 29% female) and 58.1% from non-ICU wards (64% male; 36% female). In both settings, MRSA colonized the two extremes of age (10–29 and 70+) for males and females, with different mid-life peaks or declines by gender. However, the overall demographic distribution did not differ significantly between the ICU and non-ICU groups (*p* = 0.287). Respiratory specimens constituted 37% and had the highest MRSA rate (42%), followed by blood (24.5%) and wounds (10.3%). In contrast, MSSA dominated wounds (20.3%). Overall, 73.9% were resistant to cefoxitin and cefotaxime, whereas vancomycin, linezolid, daptomycin, and tigecycline remained highly effective. Younger non-ICU patients (10–29) had higher MSSA, whereas older ICU ones showed pronounced HA-MRSA profiles. By the virtue of methicillin resistance, all MRSA were classified as multidrug resistance. Thus, MRSA colonization of the two extremes of life mostly in ICU seniors and the dominance of invasive MSSA and CA-MRSA patterns in non-ICU youth imply early age- and gender-specific adaptations of the three lineages. MRSA colonizes both ICU and non-ICU populations at extremes of age and gender specifically. High β-lactam resistance underscores the importance of robust stewardship and age- and gender-specific targeting in screening. These findings also indicate host- and organ-specificity in the sequalae of MSSA, CA-MRSA, and HA-MRSA evolutionary dynamics, emphasizing the need for continued surveillance to mitigate MRSA transmission and optimize patient outcomes in tertiary care settings.

## 1. Introduction

Methicillin-resistant *Staphylococcus aureus* (MRSA) is a major global health issue known for causing a wide range of infections from minor skin issues to severe systemic illnesses, such as sepsis [1,2]. Resistance to methicillin and other β-lactam antibiotics, including penicillins and cephalosporins, has made treatment more difficult, resulting in higher rates of illness and death, longer hospital stays, and increased healthcare costs [3,4]. This resistance, largely driven by the *mecA* gene, means that many antibiotics that were once effective are no longer working against MRSA infections [5,6]. Invasive MRSA infections cause significantly higher morbidity and mortality rates than methicillin-sensitive *S. aureus* (MSSA) infections. For example, MRSA bacteremia is associated with prolonged hospital stays, elevated healthcare costs, and roughly double the mortality risk compared to MSSA [7]. Reviews and meta-analyses have repeatedly shown that MRSA is common in many healthcare settings around the world, highlighting the need to understand both local rates as well as the mechanisms of epidemicity and spread [3,8]. For example, a meta-analysis focused on elderly care centers found a global MRSA prevalence of 14.69%, underscoring how widespread this pathogen is [8]. Moreover, the differences between hospital-associated (HA-MRSA) and community-associated (CA-MRSA) strains have become less clear, further complicating the epidemiological picture. Said et al. (2023) demonstrated a clear lineage divergence from CA-MRSA patterns in younger individuals to HA-MRSA profiles in seniors, emphasizing age-specific host-selection from a common MSSA ancestor [9].

Tertiary care hospitals play a key role in the battle against MRSA. These hospitals, which care for many high-risk patients and perform numerous invasive procedures, as well as use a large amount of antibiotics, are particularly prone to MRSA transmission [10]. Intensive care units (ICUs) within these hospitals are even more vulnerable due to the critically ill state of patients, who often have weakened immune systems, and the common use of medical devices that can serve as entry points for infection [10]. Research has shown that MRSA infection rates are usually higher in ICUs compared to non-ICU settings, indicating a need for focused interventions in these areas [11]. The severe infections caused by MRSA in tertiary care settings—including bloodstream infections, pneumonia, surgical site infections, and skin and soft tissue infections—can lead to serious health consequences and place a significant burden on both patients and the healthcare system [12].

It is essential to understand how MRSA infections vary by age and gender to develop effective prevention and control strategies [3]. Different age groups may have varying risks of MRSA due to factors like underlying health conditions, changes in immune function, and differing levels of exposure to healthcare environments. For instance, the elderly are often reported to have higher rates of MRSA-related hospitalizations [13]. Recent evidence also points to differences between genders in MRSA prevalence and outcomes [3,14]. While many studies report higher MRSA carriage and bloodstream infection rates among males [15,16], others have observed notable MRSA rates in certain female populations, underlining the complexity of these patterns. Such conflicting findings suggest that local demographic factors—ranging from behavioral to biological influences—can affect MRSA prevalence in distinct ways, necessitating further targeted research.

Studying MRSA profiles separately in ICU and non-ICU settings within tertiary hospitals is crucial because of the unique clinical environments and patient populations in these areas [17]. ICUs often have a higher rate of multidrug-resistant organisms, including MRSA, due to the factors already mentioned—critical illness, the frequent use of invasive devices, and strong selective pressure from broad-spectrum antibiotics [18]. Infection rates of healthcare-associated MRSA are higher in ICUs than in general wards [19]. Furthermore, the types of infections and patterns of antibiotic use can vary greatly between these units, which may influence the characteristics of the MRSA strains seen in each setting. Comparing MRSA profiles between these areas can uncover important differences in epidemiology and resistance patterns within the same hospital, thereby helping to tailor specific infection control measures and antibiotic guidelines.

This observational study aims to provide a detailed understanding of age- and gender-specific MRSA and CA-MRSA profiles along with their associated disease patterns, within our tertiary care hospital ICU and non-ICU settings. To do this, we used data from 253 *S. aureus* isolates of MRSA and CA-MRSA for their profiles in the two settings across different age and gender groups and describe the related disease patterns and antibiotic susceptibility profiles. Focusing on a single tertiary care hospital can generate valuable local epidemiological data that may guide targeted infection control and treatment strategies for our patient population and the specific MRSA strains present.

## 2. Materials and Methods

### 2.1. Study Design and Setting

This clinical surveillance study was conducted at King Salman Specialist Hospital (KSSH), King Khalid Hospital, Ha’il, from May 2023 to March 2025, as part of a scheduled MRSA surveillance program. The KSSH is a major referral center and has been accredited by the Saudi Central Board for Accreditation of Healthcare Institutions (CBAHI)—Ref.no. HAL/MOH/HO5/34213—along with the Ha’il Health Regional Laboratory (HHRL), which is also certified and accredited by the CBAHI—Code 2739. All the patients who presented with clinically suspected primary *Staphylococcus aureus* infections were included. Only primary MRSA infections, and not the overall admissions including acquired coinfections in cases with underlying conditions in the hospital, were considered. Secondary coinfections in patients with underlying causes are being dealt with in a different program and isolates are being compared for their ability for primary infections. However, in line with the major global efforts for MRSA eradication, we focused on all the hospital units prone to primary MRSA infection sources. All the sampling, identification, and clinical profiles were conducted according to the ISO standard protocols in place, as explained below.

### 2.2. Patient Enrollment, Sample Collection, and Definition of Clinical Categories

A total of 253 non-duplicate *S. aureus* isolates were obtained from patients of varying ages and genders. The patients were categorized into ICU or non-ICU groups based on their admission status at the time of sample collection and by age, 10–29, 30–49, 50–69, and 70+, and by gender. Those with suspected *S. aureus* infection were eligible for inclusion. Only patients with a laboratory-confirmed *S. aureus* isolate obtained from a clinical specimen, such as blood, respiratory secretions, or wound swabs, were included. However, certain exclusions were applied. Repeat isolates from the same patient or from the same infection site that exhibited identical diagnostic, genetic, or disease characteristics within a short interval (e.g., 14 days) were not considered. Additionally, samples that failed to grow on subculture or that exhibited polymicrobial contamination, or non-specific *nuc* gene profiles, were excluded. Infections were classified by specimen source (blood, respiratory, wounds, or other sites).

### 2.3. Laboratory Identification, Molecular Characterizations, and Antibiotic Susceptibility Testing

Routine bacteriological methods were employed for the initial identification of the *Staphylococcus aureus* isolates. All the clinical specimens were inoculated onto standard culture media and incubated at 37 °C for 18–24 h. Isolates showing typical colony morphology were subjected to biochemical testing and molecular confirmation by the *nuc* gene, specific for *S. aureus*. Antimicrobial susceptibility testing was performed using automated systems, namely, the BD Phoenix (BD Biosciences, Franklin Lakes, NJ, USA), VITEK 2 (bioMérieux, Marcy-l’Étoile, France), and MicroScan Plus (Beckman Coulter, Brea, CA, USA). The testing procedures and interpretive criteria were based on the standards provided by the Clinical and Laboratory Standards Institute (CLSI, M100-26). The zone diameters and minimum inhibitory concentrations (MICs) were interpreted accordingly, and where necessary, additional agar diffusion testing was performed to confirm resistance to oxacillin and other beta-lactams. Standard quality control strains, such as *S. aureus* ATCC 25923, were used consistently to ensure the accuracy and reproducibility of the results. Isolates showing phenotypic resistance to cefoxitin and/or oxacillin were classified as methicillin-resistant *S. aureus* (MRSA), in accordance with the CLSI guidelines.

To further confirm methicillin resistance and to avoid misclassification due to borderline or inducible resistance, molecular detection was conducted using the GeneXpert^®^ Dx System (Cepheid, Sunnyvale, CA, USA). This real-time PCR platform allowed for the direct detection of key genetic markers, including *mecA*, *nuc*, and *spa*, as well as *SCCmec* elements. The system utilizes an integrated, closed-cartridge format that automates sample purification, amplification, and detection, minimizing the risk of contamination and reducing bias introduced by in vitro subculturing. Direct molecular testing from clinical specimens ensured the accurate identification of MRSA strains and preserved the genetic integrity of the original isolate.

All the isolates confirmed as resistant to either cefoxitin or oxacillin and carrying the *mecA* gene were categorized as MRSA. Susceptible isolates without *mecA* were classified as methicillin-sensitive *S. aureus* (MSSA). Based on their resistance profiles, the MRSA isolates were further divided into hospital-associated MRSA (HA-MRSA) resistant to both beta-lactam and non-beta-lactam (classified as multidrug-resistant) and CA-MRSA remained susceptible to non-beta-lactam agents. The antibiotic panel tested included agents from across multiple categories: beta-lactams (cefoxitin, oxacillin, ampicillin, penicillin, and cefotaxime), fluoroquinolones (ciprofloxacin, levofloxacin, moxifloxacin, and ofloxacin), aminoglycosides (gentamicin), macrolides and lincosamides (erythromycin and clindamycin), glycopeptides (vancomycin and teicoplanin), oxazolidinones (linezolid), lipopeptides (daptomycin), tetracyclines (tetracycline and tigecycline), and other agents, including trimethoprim, nitrofurantoin, rifampicin, mupirocin, and ceftaroline. The classification of the isolates as multidrug-resistant (MDR), extensively drug-resistant (XDR), or pan-drug resistant (PDR) was performed according to the definitions established by the European Centre for Disease Prevention and Control (ECDC), as described by Magiorakos et al. [20]. These classifications were based on acquired resistance across different antimicrobial categories, excluding intrinsic resistance mechanisms.

### 2.4. Direct Multi-Gene Molecular Detection of S. aureus Lineages by GeneXpert System

The GeneXpert system employs direct molecular detection from patient specimens, providing accurate strain profiles consistent with clinical presentation, demographic data, and disease classification. This reduces artifact mutations that can significantly distort the genetic profiles of isolates originally obtained from patients [21,22]. We used the latest versions of the Cepheid GeneXpert^®^ Dx system, utilizing the SA Complete and MRSA assay kits, following the manufacturer’s protocols and kit specifications. This is a fully automated, multiplex real-time polymerase chain reaction (PCR) system with preloaded primers and probes targeting the *nuc*, *spa*, *mecA*, and *SCCmec* genes. These are housed in single-use, self-contained cartridges with built-in reagents. The system integrates sample purification, amplification, and detection into a single workflow, minimizing contamination, DNA degradation, and sample handling errors. Each cartridge is inoculated directly with the patient sample (e.g., swab) and inserted into the instrument, which is connected to a personal computer with preinstalled software for test operation and result interpretation. Quality assurance is maintained through internal controls: the Sample Processing Control (SPC) monitors adequate processing of the bacterial target and checks for potential PCR inhibitors, while the Probe Check Control (PCC) verifies reagent rehydration, PCR tube filling, probe functionality, and dye stability. These controls help ensure the reliability and accuracy of the molecular detection process.

### 2.5. Data Management and Statistical Analysis

Demographic, microbiological, and all other data were recorded using local databases and Microsoft Excel and REDCap version 11.1.29 and then imported into Statistical Package for Social Sciences software (SPSS, IBM; Version 24 for Windows (SPSS, Inc., Chicago, IL, USA). The prepared data were analyzed using descriptive stratified analysis. We present absolute numbers, proportions, and graphical distributions. We conducted Chi-square and exact statistical tests for proportions and show the *p*-values where appropriate (a *p*-value <0.05 was considered statistically significant). Prior to analysis, the continuous variables were assessed for normality using the Shapiro–Wilk test. Due to the non-normal distribution of the demographic data, non-parametric tests were applied. The differences in the demographic characteristics (age and gender distributions) between the ICU and non-ICU populations (Table 1) were evaluated using the Mann–Whitney U test. A two-tailed *p*-value < 0.05 was considered statistically significant. The categorical variables—such as the distribution of clinical specimen sources (blood, respiratory, wounds, and other sites; Table S1), MRSA versus MSSA prevalence (Tables 3 and 4), and antibiotic resistance profiles (Table 2)—were compared using Chi-square tests for independence. For each 2 × 2 contingency table (e.g., comparing MRSA prevalence between ICU and non-ICU within specific age groups), degrees of freedom were set to 1, and significance was defined as *p* < 0.05. In addition, gender-specific comparisons of MRSA prevalence across ICU and non-ICU settings were performed using Chi-square tests (as shown in the merged analysis in Table 5). All the *p*-values reported are two-tailed. Overall, these statistical methods allowed us to assess whether differences in demographic distributions, MRSA/MSSA prevalence, and antibiotic resistance patterns were statistically significant, thus supporting targeted infection control and treatment strategies.

## 3. Results

### 3.1. Demographic Distribution of S. aureus Isolates in ICU and Non-ICU Settings

A total of 106 isolates (41.9%) were recovered from the ICU, whereas 149 isolates (58.1%) were obtained from non-ICU areas. Within the ICU cohort (*n* = 106), 75 (71%) isolates originated from male patients and 31 (29%) from female patients. Among the male ICU patients, the 70+ age group had the highest proportion (28/75; 61.9%), followed by the 10–29 group (15/75; 51.7%), the 50–69 group (25/75; 43.1%), and the 30–49 group (7/75; 17.9%). For the female ICU patients, the highest proportion of isolates was observed in the 10–29 group (7/15; 46.7%), followed by 30–49 (7/19; 36.8%), 50–69 (8/33; 24.2%), and 70+ (9/18; 50.0%) (Table 1).

Among the non-ICU patients (*n* = 149), (64%; *n* = 95) isolates were obtained from males and (36%; *n* = 54) from females. The male non-ICU patients showed the highest proportion of isolates in the 30–49 age group (32/39; 82.1%), followed by 50–69 (33/58; 56.9%), 10–29 (14/29; 48.3%), and 70+ (16/42; 38.1%). For the female non-ICU patients, the highest proportion was found in the 50–69 group (25/33; 75.8%), followed by 30–49 (12/19; 63.2%), 10–29 (8/15; 53.3%), and 70+ (9/18; 50.0%) (Table 1).

A comparison of the demographic distribution between the ICU and non-ICU populations using the Mann–Whitney U test yielded no statistically significant difference (U = 6641.5, Wilcoxon W = 20,837.5, Z = –1.064, and *p* = 0.287), indicating that, while there were differences in some absolute numbers, the overall demographic patterns did not differ significantly (Table 1).

### 3.2. Distribution of Clinical Specimen Sources Among Patient Groups

**Figure 1:**
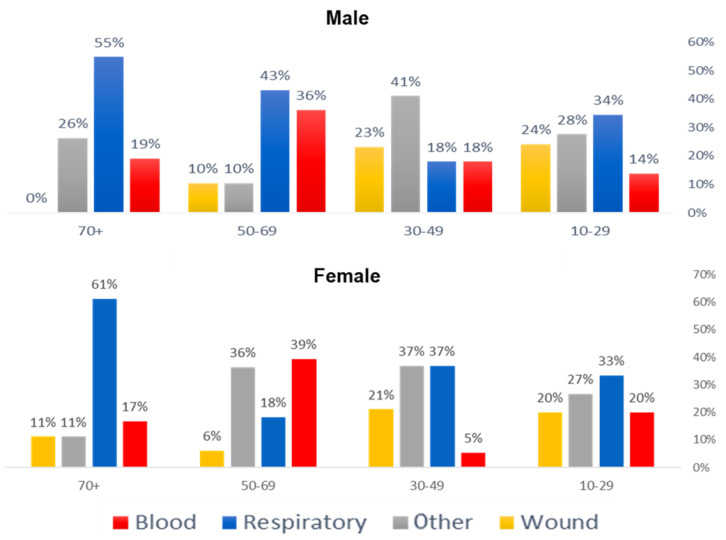
Distribution of *Staphylococcus aureus* isolates by clinical specimen source across different age groups and genders. The bar chart represents the percentage of isolates recovered from blood (red), respiratory samples (blue), other sites (gray), and wounds (yellow) among male (**top**) and female (**bottom**) patients in four age groups (70+, 50–69, 30–49, and 10–29 years).

A total of 60 isolates (24%) were derived from blood, 94 (37%) from respiratory samples, 66 (26%) from other sites, and 33 (13%) from wounds. In male patients, the 50–69 age group exhibited the highest proportion of respiratory isolates (43.1%), followed by blood samples (36.2%) and other sites (10.3%). The 70+ male group had the highest respiratory isolation rate (54.8%), with blood samples contributing 19.0% and other sites 26.2%, while no isolates were obtained from wounds. The 30–49 male group showed 41.0% of isolates from other sites, while respiratory and blood samples each accounted for 17.9%. In the 10–29 male group, respiratory samples made up 34.5%, other sites 27.6%, wound isolates 24.1%, and blood samples 13.8%. Among the female patients, the 70+ age group had the highest proportion of respiratory isolates (61.1%), while blood, wound, and other site isolates each contributed 16.7%, 11.1%, and 11.1%, respectively. The 50–69 female group had a more balanced distribution, with 39.4% of the isolates from blood, 18.2% from respiratory samples, 36.4% from other sites, and 6.1% from wounds. The 30–49 female group recorded 36.8% respiratory isolates, 36.8% from other sites, 21.1% from wounds, and 5.3% from blood. The 10–29 female group exhibited a distribution of 33.3% respiratory isolates, 26.7% from other sites, and 20.0% each from blood and wound isolates as shown in Figure 1 (Table S1). Overall, 45 blood isolates (24.5%) were identified as MRSA, compared to 15 (21.7%) MSSA. Similar trends were observed in the respiratory and wound specimens, where MRSA accounted for 41.8% and 10.3% of the isolates, respectively, while MSSA made up 24.6% and 20.3% of the isolates, respectively. The respiratory samples had the highest percentage of MRSA, while the wound samples had the lowest as shown in Figure 1 (Table S1).

### 3.3. Antibiotic Resistance Profiles of Staphylococcus aureus Isolates

A substantial proportion of isolates demonstrated resistance to β-lactam antibiotics. Specifically, 73.9% were resistant to cefoxitin and cefotaxime, while ampicillin and penicillin resistances each reached 96%. In line with the CLSI criteria and molecular confirmation (GeneXpert), any isolate with positive resistant elements and resistance to cefoxitin or oxacillin was classified as MRSA. Among these MRSA strains, many also exhibited co-resistance to non-β-lactam antibiotics, reflecting an HA-MRSA profile. In contrast, MRSA isolates that remained susceptible to most non-β-lactam agents aligned with CA-MRSA characteristics.

High fluoroquinolone resistance was observed, with 73.1% of isolates resistant to ofloxacin, whereas ciprofloxacin and levofloxacin maintained susceptibility rates of 68.0% and 69.6%, respectively. Resistance to gentamicin and trimethoprim was comparatively lower at around 20–26%. Among the glycopeptides, vancomycin and teicoplanin retained strong efficacy, with 97.6% and 96.8% susceptibility, respectively, and only rare intermediate or resistant strains were detected. Daptomycin also remained highly active, with 98.4% of the isolates susceptible.

Regarding lincosamide and macrolide agents, clindamycin resistance was noted in 22.5% of isolates (with 0.4% intermediate), and erythromycin resistance reached 34.4%. Linezolid showed excellent in vitro activity, with 99.6% of isolates susceptible. Nitrofurantoin (97.6%), rifampicin (98.4%), and tetracycline (88.9%) also exhibited high activity, whereas moxifloxacin susceptibility was 68.4%. Ceftaroline and tigecycline both displayed favorable profiles (95.7% and 98.4% susceptibility, respectively) (Table 2 and Supplementary Materials Figure S1).

Taken together, these findings highlight the predominance of MRSA in this setting, driven by broad β-lactam resistance and, in many cases, co-resistance to fluoroquinolones. However, vancomycin, daptomycin, linezolid, and tigecycline remained effective therapeutic options.

### 3.4. Age- and Gender-Specific Prevalence of MRSA and MSSA

Among the male patients (*n* = 168), 117 isolates (69.6%) were identified as MRSA and 51 (30.4%) as MSSA. In contrast, among the female patients (*n* = 85), 67 isolates (78.8%) were MRSA and 18 (21.2%) were MSSA. Within the ICU setting, the highest proportion of MRSA was observed in the 10–29 age group (20/22; 90.9%), followed by the 50–69 group (29/33; 87.9%). The 30–49 age group had a MRSA prevalence of 64.3% (9/14). The patients aged 70+ in the ICU showed a MRSA proportion of 68.6% (24/35), remaining more frequently MRSA than MSSA. In non-ICU settings, the distribution of MRSA differed notably across age groups. The 10–29 age group exhibited 45.5% MRSA (10/22), which was the only category in which MSSA was more prevalent (54.5%). The 30–49 group had a MRSA prevalence of 59.1% (26/44), whereas the 50–69 age group demonstrated a 79.3% (46/58) MRSA prevalence. In the 70+ non-ICU group, MRSA accounted for 80.0% (20/25) of the isolates, the highest proportion outside the ICU (Table 3). Taken together, these findings highlight a pronounced prevalence of MRSA among both male and female patients, with particularly high MRSA proportions in middle-aged and older adults across ICU and non-ICU settings. The 10–29 non-ICU group showed a different trend, where MSSA exceeded MRSA prevalence, suggesting potential differences in community-acquired versus hospital-acquired infection patterns in younger patients. These findings emphasize the need for targeted infection control measures and antibiotic stewardship programs in both ICU and general hospital wards (Table 3).

### 3.5. Gender-Based Differences in MRSA and MSSA Prevalence Across ICU and Non-ICU Settings

When analyzing gender distribution by age group in ICU and non-ICU settings, notable differences emerged between the male and female patients. In the 10–29 age group, non-ICU females (64.0%) had a higher MRSA rate than ICU females (36.0%), while the rates in males were similar between ICU (51.7%) and non-ICU (48.3%). In the 30–49 and 50–69 age groups, the non-ICU patients, especially females, had higher MRSA prevalence than the ICU patients. Among the 70+ patients, the MRSA rates were evenly distributed between the ICU and non-ICU settings (50.0%) in females, while the ICU males had a higher MRSA prevalence (67.0%) compared to the non-ICU males (33.0%). These findings highlight age- and gender-specific MRSA trends, emphasizing the need for tailored infection control strategies in both ICU and non-ICU settings (Table 4).

A 2 × 2 Chi-square test was performed for each age group to compare the gender distribution (male vs. female) between the ICU and non-ICU settings. Each 2 × 2 table had one degree of freedom. A significant difference (*p* < 0.05) was observed only in the 50–69 age group (X^2^ = 10.766; *p* = 0.001), indicating that males in this bracket were more likely to be in the ICU, whereas females were more frequently in the non-ICU group. In the 10–29, 30–49, and 70+ age groups, there was no significant difference in the male/female distribution (*p* > 0.05). Thus, 50–69 stands out as the only age range with a statistically meaningful discrepancy in gender distribution across ICU and non-ICU settings (Table 5).

## 4. Discussion

*Staphylococcus aureus* continues to evolve and emerge as a strong adversary in healthcare, especially in large hospitals where complex patient conditions and invasive procedures help it spread. We have provided significant insights into the distributions of *S. aureus* lineages across different patient demographics and hospital units, as well as their antimicrobial susceptibility and resistance patterns. We provide important clinical implications towards MRSA frequencies, management, and control practices. The gender-specific respiratory colonization of MRSA at the two extremes of age and the dominance of MSSA in cutaneous infection in youth underscores the need for age-specific screening protocols. These findings, along with genotypic characterizations, multidrug resistance MRSA, and non-beta-lactam susceptibility patterns, indicate the need for specific patient treatment and management strategies, robust stewardship programs, and age- and gender-specific profiling of lineages. These findings also indicate host- and organ-specificity of MSSA, CA-MRSA, HA-MRSA dynamics, emphasizing the need for continued surveillance to mitigate MRSA transmission and optimize patient outcomes in tertiary care settings.

Our study of 253 *S. aureus* isolates (41.9% from ICU; 58.1% from non-ICU), which examined the age- and gender-specific presence and disease profiles of MRSA and MSSA, provided important trends supporting and expanding on earlier reports in this field. A key part of MRSA’s challenge in the clinic is its resistance to β-lactam antibiotics, driven by the *mecA* gene [23]. This change makes common treatments, such as penicillins and cephalosporins, mostly ineffective and forces clinicians to use other alternatives, including glycopeptides and oxazolidinones [24]. Our results, showing 73.9% resistance to cefoxitin/cefotaxime and 96% resistance to ampicillin/penicillin, mirrored these well-known mechanisms and echoed global reports that highlight the growing difficulties in treating MRSA infections [25,26]. Nevertheless, the major pitfall in *S. aureus* treatment and management strategies has been the mere reliance on new drugs alone, juxtaposing the species’ potential similar to that of all other bacteria. As we and others have been examining for decades, the widely known rapid adaptational capacity of the species and the evolution of resistant clones despite genome-stability and clonal backgrounds in humans and animals [22,27] makes it imperative for a relevant change in the MRSA control strategy. This species is one of the most powerful pathogens ever known, expressing subtle human-specialized mechanisms of adaptations and genome-plasticity [28,29]. It is therefore plausible that the age, gender, ecosystems, and host microenvironments all differentiate the evolution of clonal lineages specific to each demographic factor [30,31]—and these should be the major focus for the Superbug. The high proportion of MRSA in our hospital, across both ICU and non-ICU settings, reminds us that MRSA is still firmly rooted in areas where the risk is elevated. In our ICU population, which comprised 71% male and 29% female isolates, invasive procedures and intensive antibiotic pressure create an environment that favors MRSA persistence [32,33]. Many studies have observed that ICU patients face higher MRSA rates than those in general wards, a fact that our data also reflected [34,35].

Our findings show that while patient characteristics between the ICU and non-ICU groups did not differ greatly, there were distinctions in the absolute numbers and infection types. Older ICU patients, particularly those aged 70+ years, experienced more respiratory (54.8% in males and 61.1% in females) and bloodstream infections, likely reflecting ventilator use and immunocompromised status. This is in line with work showing that critically ill patients often develop severe MRSA infections, such as bacteremia and pneumonia, which can lead to higher illness and death rates [36,37,38].

When we looked at age-specific trends, we saw that MRSA became more common in older individuals. This pattern is well documented in many places: older adults, particularly those above 60, are more prone to MRSA infection due to factors like a weaker immune system and more chronic diseases [14,39,40]. In our data, the 70+ group had high MRSA rates in both ICU and non-ICU wards. Meanwhile, younger people also develop MRSA, though their patterns can differ. Some works describe a “J-shaped” curve where both very young and older patients carry higher MRSA rates [9,35]. This likely points to different causes in children and older adults. Among our younger patients (10–29 years) in non-ICU areas, MSSA sometimes matched or exceeded MRSA rates, suggesting that these are evolutionary ancestors for invasive skin and soft tissue infections emerging as CA-MRSA in youth and HA-MRSA by age [41].

We also observed notable gender differences in MRSA rates. While many reports find that men are more likely to have MRSA—attributed to factors like greater colonization and different hygiene practices [3,42]—our work showed that some female groups had higher MRSA levels. Conflicting results in various studies emphasize that gender-related differences in MRSA likely arise from local, behavioral, and even hormonal issues [43,44]. For instance, a study in a maternity and children’s hospital found increased MRSA in young female patients, suggesting that local circumstances and certain risks can outweigh the usual male dominance [43]. In our data, young female patients in the ICU had lower MRSA rates than those outside the ICU, while older groups had fewer differences. This suggests that gender-related risks might blend with clinical settings to form unique local patterns of MRSA.

Our review of diseases tied to MRSA and MSSA offers more insights into their clinical impact. Many reports point to skin and soft tissue infections (SSTIs) as the main expression of MRSA, especially in the community [40,45,46]. While SSTIs were common across ages in our findings, older and ICU patients more often had respiratory and bloodstream infections. This is logical, as older or critically ill individuals may have weaker lungs or require devices, raising their risk of serious infections. We also saw that younger patients are more likely to present with SSTIs, while older groups are more prone to pneumonia and bacteremia. These differences show how diagnosis and treatment might need to be customized by age and setting.

Our detailed look at antibiotic resistance patterns is another vital piece. MRSA isolates in our study largely resisted β-lactams and fluoroquinolones, reflecting the effect of the *mecA* gene and pressures from antibiotic use [47]. However, they remained mostly vulnerable to glycopeptides (such as vancomycin), oxazolidinones (such as linezolid), and newer treatments, like tigecycline and daptomycin. These data are in line with several studies from similar hospitals, which show that, although older antibiotics are losing ground, the newer “last-resort” agents still work [9,40,48]. Nonetheless, the strong resistance to fluoroquinolones in our study is concerning since these drugs are often used first in practice. Differences in resistance patterns between our data and those from places like Vietnam and India stress the need for stewardship programs that watch local trends [3,44].

When comparing ICU and non-ICU wards, we found that although there were no huge differences in overall patient demographics, the ICU environment still carried a heavier load of severe MRSA infections. ICUs are known hotspots because of high-risk patients and the frequent use of invasive devices and broad-spectrum antibiotics [10]. Our data, showing greater MRSA rates in ICU patients (especially in bloodstream and lung infections), strengthen this well-recognized situation. It also signals the importance of strong infection control in ICUs, including active screening, careful hand hygiene, and decolonization. Older adults in the ICU were hit the hardest by MRSA, reinforcing the idea that they deserve extra attention in control measures.

Local patterns do not exist in a vacuum, and our study fits into global observations. Worldwide, MRSA prevalence can vary widely, from around 13% in some places to over 70% in others [49,50]. For example, Saudi Arabia’s average MRSA rate is about 32.5%, and it has been rising [43]. Meanwhile, certain European nations, such as the Netherlands, have kept rates very low with strong control efforts [51]. Our hospital’s MRSA and MSSA levels must be interpreted in this broad context, where health policies, antibiotic usage, and population traits differ. By offering a careful snapshot of MRSA in a tertiary hospital, our study reminds us that even within one place, age, gender, and clinical areas can shape different MRSA patterns.

These insights have several implications. Clinically, the high MRSA rate in older age groups and in the ICU suggests that initial antibiotic choices in these groups should cover MRSA. With β-lactams and fluoroquinolones often failing, clinicians should consider drugs proven effective against MRSA, such as vancomycin or linezolid. From an infection control view, our data hint that screening and decolonizing might be especially effective in the ICU and among older patients. For example, swabbing for MRSA at admission and then isolating and decolonizing those who test positive might lower the spread in these high-risk settings.

Regarding gender-based trends, our findings reveal that while some works describe a higher burden in men, local factors may create a different picture. This indicates that prevention and treatment plans for MRSA should be flexible and consider the specific patient groups served by each hospital [10,44]. Future research could focus on the social, biological, and cultural elements behind these gender differences, paving the way for more personal interventions.

Lastly, our review of antibiotic susceptibility underscores how quickly resistance patterns can shift. The marked resistance to widely used drugs in our study underlines the need for steady local observation of resistance and for strict antibiotic stewardship programs. As patterns change, it is crucial that providers adjust their standard therapies and that infection control teams track these changes constantly. With the threat of resistance to even our newest treatments, we must keep searching for fresh therapies and different approaches to fighting MRSA.

In summary, our work offers a clear view of how MRSA and MSSA lineages differ by age, gender, and disease type in a tertiary hospital, relevant to host- and tissue-specific evolutions. The strong presence of MRSA in older adults and ICU settings, contrasted with community-associated patterns among younger non-ICU patients, underscores the need for targeted interventions and tailored treatment strategies. These findings are immediately relevant to everyday practice, highlighting the significance of host-specialized screening, relevant infection control, and updated antibiotic policies to ease the burden of MRSA in high-risk areas.

In the bigger picture, we confirm that while our local trends mirror many of the global patterns—such as older adults and ICU patients being more prone to MRSA—our hospitals also have unique details that deserve further study. The next steps should involve molecular and genomic research to track MRSA’s ancestor lines in our setting and discover potential connections between strains from the community and the hospital. Such future work would deepen our understanding of MRSA’s spread and lead to even more precise infection control.

## 5. Conclusions

In conclusion, our study emphasizes the urgent need for careful MRSA management strategies toward host-specialized lineages in tertiary care facilities. MRSA colonization at the two extremes of life, mostly in ICU seniors, and the dominance of invasive MSSA and CA-MRSA patterns in non-ICU youth imply age-specific screening programs are beneficial. High β-lactam resistance underscores the importance of robust stewardship programs. Ongoing surveillance, targeted screening for lineage ancestors in youth, and strong antibiotic stewardship remain essential to mitigate the clinical and economic impacts of MRSA in hospital environments. The use of direct molecular detection from specimens, age- and gender-specific screening, and phenotypic and genotypic resistance classifications are the most important strengths of this study. These minimize mutations, sustain lineage genotypic properties, and allow for resistance pattern comparisons and confirmations. However, this study is limited by the focus on a few centers and the number of samples. While it is useful in giving only a local strain profile, it does not represent the breadth of the species’ diversity. Future large-scale molecular surveillance and sequencing would gain more insights into the mechanisms of adaptive evolution and emergence of *S. aureus* lineages.

## Figures and Tables

**Table 1:** Demographic distribution of *S. aureus* isolates in ICU and non-ICU settings, stratified by age group and gender.

ICU Status	Gender		Age Group	Count	% Within Group
ICU	Male		10–29	15	(51.70%) (*n* = 29; 15ICU + 14non ICU)
			30–49	7	(17.90%) (*n* = 39; 7 ICU + 32non ICU)
			50–69	25	(43.10%) (*n* = 58; 25ICU + 33non ICU)
			70+	28	(63.90%) (*n* = 44; 28ICU + 16non ICU)
	Subtotal Male (ICU)		75		
	Female		10–29	7	(46.70%) (*n* = 15; 7ICU + 8nonICU)
			30–49	7	(36.80%) (*n* = 19; 7ICU + 12nonICU)
			50–69	8	(24.20%) (*n* = 33; 8ICU + 25nonICU)
			70+	9	(50.00%) (*n* = 18; 9ICU + 9nonICU)
	Subtotal Female (ICU)	31			
	Total ICU			106	
Non-ICU	Male		10–29	14	(48.30%) (*n* = 29; 15ICU + 14non ICU)
			30–49	32	(82.10%) (*n* = 39; 7 ICU + 32non ICU)
			50–69	33	(56.90%) (*n* = 58; 25ICU + 33non ICU)
			70+	16	(38.10%) (*n* = 44; 28ICU + 16non ICU)
	Subtotal Male (Non-ICU)		95		
	Female		10–29	8	(53.30%) (*n* = 15; 7ICU + 8nonICU)
			30–49	12	(63.20%) (*n* = 15; 7ICU + 8nonICU)
			50–69	25	(75.80%) (*n* = 15; 7ICU + 8nonICU)
			70+	9	(50.00%) (*n* = 18; 9ICU + 9nonICU)
	Subtotal Female (Non-ICU)			54	
	Total Non-ICU			149	
Overall Total	Male			168	
	Female			85	
	Grand Total			253	
Statistical Analysis Results Comparing ICU and Non-ICU Groups					
Test Statistic			Value		
Mann–Whitney U			6641.5		
Wilcoxon W			20,837.5		
Z			−1.064		
Asymp. Sig. (2-tailed)			0.287		

**Table 2:** Antibiotic resistance patterns of *S. aureus* isolates, MRSA and MSSA.

	Resistant	Susceptible	Intermediate
Antibiotics (Chemical Group)	N% (*n*=)	N% (*n*=)	N% (*n*=)
Cefoxitin (second-generation cephalosporins)	73.90% (*n* = 187)	26.10% (*n* = 66)	0.00% ( *n* = 0)
Cefotaxime (third-generation cephalosporin)	73.90% (*n* = 187)	26.10% (*n* = 66)	0.00% (*n* = 0)
Ampicillin (β-lactam)	95.70% (*n* = 242)	1.60% (*n* = 4)	2.80% (*n* = 7)
Penicillin (β-lactam)	96.00% (*n* = 243)	1.20% (*n* = 3)	2.80% (*n* = 7)
Ofloxacin (quinolone)	73.10% (*n* = 185)	26.90% (*n* = 68)	0.00% (*n* = 0)
Gentamicin (aminoglycoside)	26.50% (*n* = 67)	73.50% (*n* = 186)	0.00% (*n* = 0)
Trimethoprim (diaminopyrimidines)	20.60% (*n* = 52)	79.40% (*n* = 201)	0.00% (*n* = 0)
Teicoplanin (glycopeptide)	2.80% (*n* = 7)	96.80% (*n* = 245)	0.40% (*n* = 1)
Vancomycin (glycopeptide)	0.40% (*n* = 1)	97.60% (*n* = 247)	1.20% (*n* = 3)
Clindamycin (lincosamide)	22.50% (*n* = 57)	77.10% (*n* = 195)	0.40% (*n* = 1)
Erythromycin (macrolide)	34.40% (*n* = 87)	65.20% (*n* = 165)	0.40% (*n* = 1)
Linezolid (oxazolidinone)	0.40% (*n* = 1)	99.60% (*n* = 252)	0.00% (*n* = 0)
Nitrofurantoin (nitrofuran)	0.40% (*n* = 1)	97.60% (*n* = 247)	2.00% (*n* = 5)
Moxifloxacin (fluoroquinolone)	26.50% (*n* = 67)	68.40% (*n* = 173)	5.10% (*n* = 13)
Rifampicin (rifamycin)	1.20% (*n* = 3)	98.40% (*n* = 249)	0.40% (*n* = 1)
Tetracycline (broad spectrum)	10.70% (*n* = 27)	88.90% (*n* = 225)	0.40% (*n* = 1)
Ciprofloxacin (quinolone)	31.60% (*n* = 80)	68.00% (*n* = 172)	0.40% (*n* = 1)
Mupirocin (carboxylic acid)	3.60% (*n* = 9)	96.40% (*n* = 244)	0.00% (*n* = 0)
Daptomycin (cyclic lipopeptides)	0.00% (*n* = 0)	98.40% (*n* = 249)	1.60% (*n* = 4)
Ceftaroline (cephalosporin)	4.00% (*n* = 10)	95.70% (*n* = 242)	0.40% (*n* = 1)
Tigecycline (glycylcycline class)	0.00% (*n* = 0)	98.40% (*n* = 249)	1.60% (*n* = 4)
Levofloxacin (third generation of fluoroquinolones	21.70% (*n* = 55)	69.60% (*n* = 176)	8.70% (*n* = 22)

**Table 3:** Age and gender distribution of MRSA and MSSA isolates in ICU and non-ICU settings.

Category			MRSA	MSSA
Male	Count		117	51
	N%		69.6%	30.4%
Female	Count		67	18
	N%		78.8%	21.2%
ICU	10–29	Count	20	2
		N%	90.9%	9.1%
	30–49	Count	9	5
		N%	64.3%	35.7%
	50–69	Count	29	4
		N%	87.9%	12.1%
	70+	Count	24	11
		N%	68.6%	31.4%
Non-ICU	10–29	Count	10	12
		N%	45.5%	54.5%
	30–49	Count	26	18
		N%	59.1%	40.9%
	50–69	Count	46	12
		N%	79.3%	20.7%
	70+	Count	20	5
		N%	80.0%	20.0%

**Table 4:** Comparison of MRSA prevalence between male and female patients in ICU and non-ICU setting by age groups.

	Sub-Category		Male N (ICU vs. Non-ICU%)	Female N (ICU vs. Non-ICU%)
Age Group (ICU vs. non-ICU)	10–29 years	ICU	15 (51.7%)	9 (36.0%)
		Non-ICU	14 (48.3%)	16 (64.0%)
	30–49 years	ICU	7 (17.9%)	7 (36.8%)
		Non-ICU	32 (82.1%)	12 (63.2%)
	50–69 years	ICU	25 (64.1%)	8 (25.0%)
		Non-ICU	14 (35.9%)	24 (75.0%)
	70+ years	ICU	26 (67.0%)	9 (50.0%)
		Non-ICU	13 (33.0%)	9 (50.0%)

**Table 5:** Statistical comparison of infection distribution across ICU and non-ICU settings using a 2 × 2 Chi-square test for each age group.

Age Group	ICU (M, F)	Non-ICU (M, F)	X^2^	*p*-Value	Significance
10–29	(15, 9)	(14, 16)	1.344	0.246	ns
30–49	(7, 7)	(32, 12)	2.522	0.112	ns
50–69	(25, 8)	(14, 24)	10.766	0.001	Yes
70+	(26, 9)	(13, 9)	1.439	0.23	ns

## Data Availability

The original contributions presented in this study are included in the article/Supplementary Materials. Further inquiries can be directed to the corresponding author.

## Supplementary Materials

The following supporting information can be downloaded at: https://www.mdpi.com/article/10.3390/microorganisms13051078/s1, Table S1. Distribution of Staphylococcus aureus isolates by clinical specimen source, stratified by age group and gender. Figure S1. Antibiotic susceptibility profiles of Staphylococcus aureus isolates.
