# Supplementary material for: MRSA Profiles Reveal Age- and Gender-Specificity in a Tertiary Care Hospital: High Burden in ICU Elderly and Emerging Community Patterns in Youth
Source: Microorganisms. 2025 May 6;13(5):1078. doi: 10.3390/microorganisms13051078 (PMC12113950; doi:10.3390/microorganisms13051078)
Supplement: Supplementary file 1 [file microorganisms-13-01078-s001.zip › microorganisms-3578978-supplementary.pdf]

Supplementary Material:

Table S1. Distribution of *Staphylococcus aureus* isolates by clinical specimen source, stratified by age group and gender.

Attribute			Blood	Respiratory	Other	Wound
Frequency			60	94	66	33
Percent			24	37	26	13
Male	10-29 (*n*=29)	Count	4(13.80%)	10(34.50%)	8(27.60%)	7(24.10%)
	30-49 (*n*=39)	Count	7(17.90%)	7(17.90%)	16(41.00%)	9(23.10%)
	50-69(*n*=58)	Count	21(36.20%)	25(43.10%)	6(10.30%)	6(10.30%)
	70+(*n*=42)	Count	8(19.00%)	23(54.80%)	11(26.20%)	0(0.00%)
Female	10-29(*n*=15)	Count	3(20.00%)	5(33.30%)	4(26.70%)	3(20.00%)
	30-49(*n*=19)	Count	1(5.30%)	7(36.80%)	7(36.80%)	4()21.10%
	50-69(*n*=33)	Count	13(39.40%)	6(18.20%)	12(36.40%)	2(6.10%)
	70+(*n*=18)	Count	3(16.70%)	11(61.10%)	2(11.10%)	2(11.10%)
	MRSA Count(*n*-184)		45(24.50%)	77(41.80%)	43(23.40%)	19(10.30%)
	MSSA Count *(n=69)*		15(21.70%)	17(24.60%)	23(33.30%)	14(20.30%)

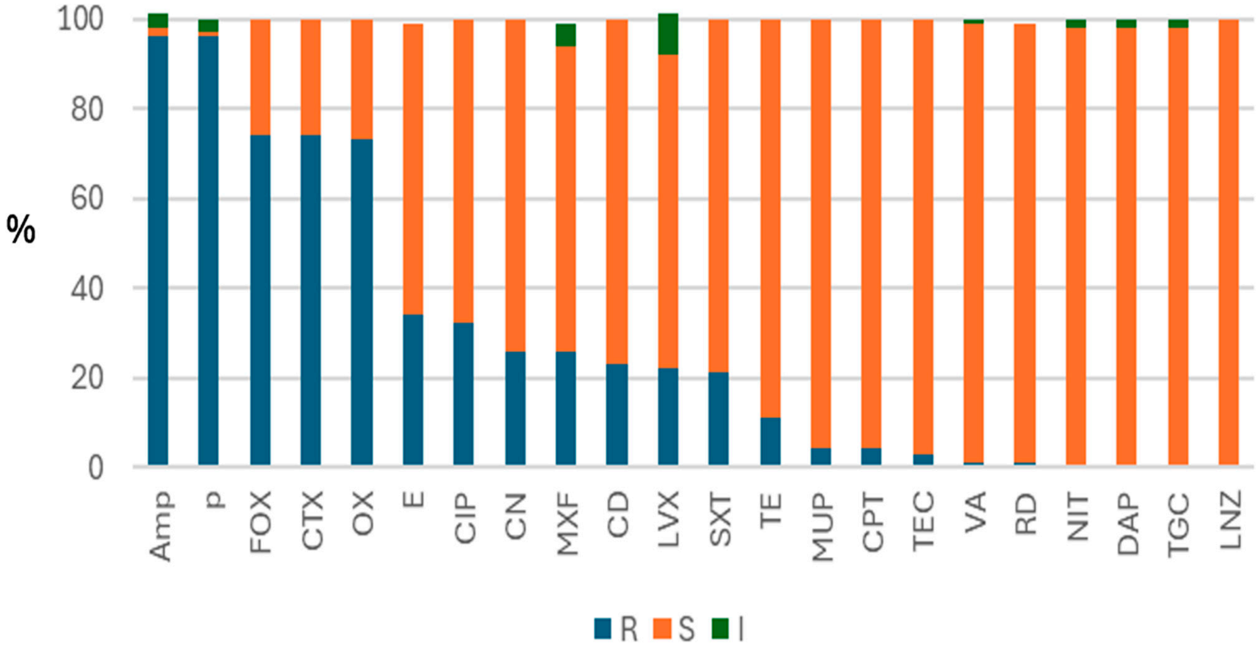

Figure S1. Antibiotic susceptibility profiles of Staphylococcus aureus isolates. The x-axis lists the tested antibiotics, abbreviated as follows: Amp (ampicillin), P (penicillin), FOX (cefoxitin), CTX (cefotaxime), OX (oxacillin), CIP (ciprofloxacin), E (erythromycin), CN (gentamicin), MXF (moxifloxacin), CD (clindamycin), LVX (levofloxacin), SXT (trimethoprim-sulfamethoxazole), TE (tetracycline), MUP (mupirocin), CPT (ceftaroline), TEC (teicoplanin), VA (vancomycin), RD (rifampicin), NIT (nitrofurantoin), DAP (daptomycin), TGC (tigecycline), and LNZ (linezolid). The y-axis indicates the percentage of isolates categorized as resistant (R, blue), susceptible (S, orange), or intermediate (I, green). Resistance to cefoxitin or oxacillin defined methicillin-resistant *S. aureus*

(MRSA), whereas strains susceptible to these agents were classified as methicillin-sensitive S. aureus (MSSA).
